# BeeKeeper 2.0: Confidential Blockchain-Enabled IoT System with Fully Homomorphic Computation

**DOI:** 10.3390/s18113785

**Published:** 2018-11-05

**Authors:** Lijing Zhou, Licheng Wang, Tianyi Ai, Yiru Sun

**Affiliations:** State Key Laboratory of Networking and Switching Technology, Beijing University of Posts and Telecommunications, Beijing 100876, China; zhoulj@bupt.edu.cn (L.Z.); aitianyi@bupt.edu.cn (T.A.); syr_2015@bupt.edu.cn (Y.S.)

**Keywords:** IoT, blockchain, outsourcing computation, publicly verifiable secret sharing, full homomorphism

## Abstract

Blockchain-enabled Internet of Things (IoT) systems have received extensive attention from academia and industry. Most previous constructions face the risk of leaking sensitive information since the servers can obtain plaintext data from the devices. To address this issue, in this paper, we propose a decentralized outsourcing computation (DOC) scheme, where the servers can perform fully homomorphic computations on encrypted data from the data owner according to the request of the data owner. In this process, the servers cannot obtain any plaintext data, and dishonest servers can be detected by the data owner. Then, we apply the DOC scheme in the IoT scenario to achieve a confidential blockchain-enabled IoT system, called BeeKeeper 2.0. To the best of our knowledge, this is the first work in which servers of a blockchain-enabled IoT system can perform any-degree homomorphic multiplications and any number of additions on encrypted data from devices according to the requests of the devices without obtaining any plaintext data of the devices. Finally, we provide a detailed performance evaluation for the BeeKeeper 2.0 system by deploying it on Hyperledger Fabric and using Hyperledger Caliper for performance testing. According to our tests, the time consumed between the request stage and recover stage is no more than 3.3 s, which theoretically satisfies the production needs.

## 1. Introduction

With the emergence of the Internet of Things (IoT), innovative applications are rapidly increasing [1]. Current IoT systems generally consist of designated lightweight devices with sensors. The devices collect data from the surrounding environment and exchange data with other devices, servers or platforms. In traditional IoT systems, the collected data are stored by centralized cloud servers, and users must trust these servers. Despite the indisputable benefits provided by these services, these centralized IoT systems face the following potential issues:

- The data stored by servers have a risk of being modified or deleted by the servers.
- In most systems, users have to reveal sensitive data (such as health, behavior, identity and private life information) to the servers [2] since the servers can obtain the plaintext data.
- A huge volume of data streams will be produced by IoT devices at high speeds in the near future [3]. However, centralized servers are neither sufficiently scalable nor sufficiently efficient to address the enormous amounts of data.

Blockchain [4], which was first proposed by Bitcoin [5], may be a solution to the above issues of centralized IoT systems due to blockchain’s properties, such as tamper-resistance, confidentiality and decentralization. Moreover, blockchain offers the functionality of smart contracts [6], which are programs recorded on the blockchain that can be triggered by events. Thereby, smart contracts can assist devices in being more intelligent since the behavior of an IoT device can be specified by a set of smart contracts.

Currently, blockchain-enabled IoT systems are a popular research area. However, most previous constructions have not solved the issue of leaking sensitive information to servers since servers can obtain plaintext data from devices. Furthermore, in some previous constructions, although servers can only obtain encrypted data, they cannot perform homomorphic computations on encrypted data from devices. Previous studies focused on five main aspects: data sharing [7,8], data storage [9,10], access control [11,12,13], smart IoT [1,14,15,16,17] and edge computing [18,19].

Recently, in [20], we proposed a confidential blockchain-enabled IoT system called BeeKeeper 1.0, where servers cannot decrypt any encrypted data from devices but can perform homomorphic computations on the encrypted data. Consequently, although BeeKeeper 1.0 addressed the issue of leaking plaintext data to servers, it has the following limitations:

- Servers can perform only one-degree homomorphic multiplication and any number of homomorphic additions on encrypted data from a device.
- Pairing (an expensive computation) [21] is used to verify the correctness of responses sent by the servers.
- Only one device can send encrypted data and requests to the servers.

In this paper, we further study the confidential blockchain-enabled IoT system and propose a novel construction, BeeKeeper 2.0, which is much more functional and efficient than BeeKeeper 1.0 [20]. Specifically, BeeKeeper 2.0 has the following improvements:

- Servers can perform any-degree (k>2) homomorphic multiplications and any number of homomorphic additions on encrypted data from devices.
- Pairing is not used to verify the correctness of the responses of the servers.
- Devices can automatically start or restart the service protocol.
- Each device can send encrypted data and requests to the servers. In this way, each device can share its data with other devices by sending encrypted data to the servers, and each device can use the data shared by all devices by sending requests to servers.
- With more and more peers of blockchain willing to act as servers, the overall processing power of the system will gradually increase.

A basic instance of BeeKeeper 2.0 includes a group of devices, servers and validators of the blockchain. The servers can help devices process encrypted data from other devices according to requests of the devices without leaking plaintext data to the servers. The devices and servers communicate with each other by using transactions of the blockchain. The validators of BeeKeeper 2.0 are almost the same as the validators of other blockchain platforms. The only difference is that the validators of BeeKeeper 2.0 have to check the validity of commitments (e.g., signature, verification key and commitments of secret data) included in the transaction payload, in addition to general verifications. Only transactions accepted by the validators can be recorded in the blockchain.

For the BeeKeeper 2.0 system, blockchain is necessary since it provides the following benefits:

- Tamper-resistance. All verification algorithms use the verification key (VK) to check the validity of the data, so the validity and tamper-resistance of the VK should be guaranteed. Fortunately, storing the VK in the blockchain is a suitable solution. On the one hand, due to blockchain’s tamper-resistance, once data have been recorded in the blockchain, the data can be seen as immutable. On the other hand, the blockchain validators can help peers verify the VK before it is recorded in the blockchain, and only the VK accepted by the validators can be recorded. Consequently, once a VK has been recorded in the blockchain, it can be seen as valid and immutable.
- Validators of the blockchain help devices and servers verify publicly verifiable data. In BeeKeeper 2.0, most data (e.g., verification key, commitments of core-shares and commitments of responses) are publicly verifiable. If these data can be verified by some trusted party and only data accepted by the trusted party can be seen by others, the devices and servers would not need to verify them. Fortunately, in the blockchain network, all publicly verifiable transaction data can be verified by the validators before these transactions are recorded in the blockchain, and only transactions accepted by the validators can be recorded. Consequently, the validators can help devices and servers verify publicly verifiable data. In this way, the verification computation of devices and servers can be substantially reduced.

**Our contributions.** In summary, the contributions of this paper are as follows:

1. We propose a decentralized outsourcing computation (DOC) scheme based on blockchain, where servers can perform any-degree homomorphic multiplications and any number of homomorphic additions on encrypted data from the data owner. In the scheme, the servers cannot obtain any plaintext data, and if a server computes dishonestly, it will be detected by the validators of the DOC scheme and the data owner. To the best of our knowledge, this is the first work on a decentralized outsourcing computation scheme.
2. By applying the DOC scheme in the IoT scenario, we achieve a confidential blockchain-enabled IoT system with fully homomorphic computation, called BeeKeeper 2.0. We instantiate all building blocks of BeeKeeper 2.0. To the best of our knowledge, this is the first work in which: (i) devices can share their data with each other; (ii) servers can perform any-degree homomorphic multiplications and any number of additions on encrypted data from devices according to the requests of the devices; (iii) servers cannot obtain any plaintext data; and (iv) dishonest servers can be detected by the validators of the blockchain and the devices.
3. By using Hyperledger Fabric blockchain as the carrier of the BeeKeeper 2.0 system, a detailed performance evaluation of BeeKeeper 2.0 was obtained. Moreover, we used Hyperledger Caliper for performance testing. According to our tests, the time cost between the request stage and the recover stage is no more than 3.3 s, which theoretically meets the production needs.

## 2. Overview of BeeKeeper 2.0

By applying the DOC scheme, which is presented in Section 4, in the IoT scenario, we construct BeeKeeper 2.0, a confidential blockchain-enabled IoT system with fully homomorphic computation. BeeKeeper 2.0 can function on top of any blockchain platform (e.g., Bitcoin [5], Ethereum [6] and Hyperledger Fabric [22]).

**Figure 1:**
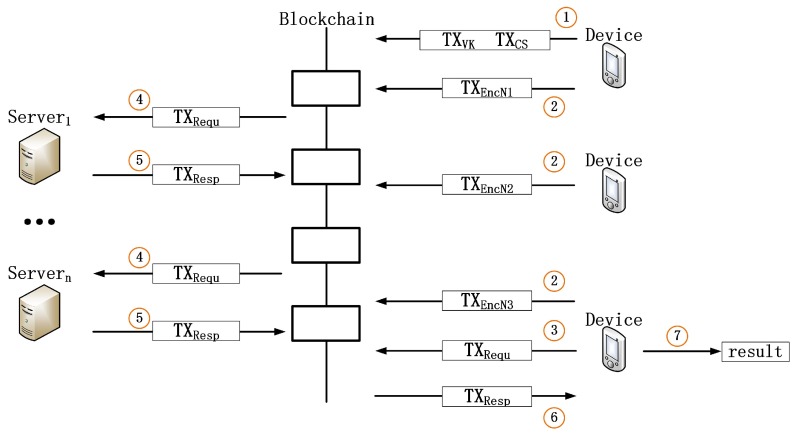
An overview of BeeKeeper 2.0. TX${}_{VK}$, TX${}_{CS}$, TX${}_{EncN}$, TX${}_{Requ}$ and TX${}_{Resp}$ are transactions sent to blockchain. TX${}_{VK}$ includes verification key (VK); TX${}_{CS}$ includes encrypted core-shares and commitments of these core-share; TX${}_{EncN}$ includes encrypted numbers; TX${}_{Requ}$ includes a request; and TX${}_{Resp}$ includes an encrypted response and a commitment of this response. This figure shows the work process of BeeKeeper 2.0 in the case where all sent transactions are valid. In fact, if any transaction is invalid, this transaction will not be appended in the blockchain, and it will not be seen by others.

A basic instance of the BeeKeeper 2.0 system includes a group of devices, a certain number of servers and validators of the blockchain. The devices and servers are peers of the blockchain. Any peer can become a server of devices as long as the peer and owner of the devices desire. The validators are responsible for verifying all transactions before these transactions are recorded in the blockchain, and only transactions accepted by the validators can be recorded. The validators of BeeKeeper 2.0 are almost the same as the validators of other blockchain platforms. The only difference is that the validators of BeeKeeper 2.0 have to check the validity of commitments included in the transaction payload, in addition to general verifications. An overview of BeeKeeper 2.0 is shown in Figure 1.

A BeeKeeper 2.0 system consists of the following algorithms:

(Setup, EncNum, Request, Respond, Recover, VerifyTx, CheckEnc).

The system also includes five new transactions: TX${}_{VK}$, TX${}_{CS}$, TX${}_{EncN}$, TX${}_{requ}$ and TX${}_{Resp}$. Specifically, TX${}_{VK}$ includes a verification key (VK); TX${}_{CS}$ includes encrypted core-shares (each encrypted core-share is encrypted by using a different server’s public-key) and commitments of core-shares; TX${}_{EncN}$ includes encrypted numbers; TX${}_{Requ}$ includes a request; and TX${}_{Resp}$ includes an encrypted response (the response is encrypted by using a device’s public-key) and a response’s commitment.

In this paper, we hope to complete a scenario as follows:

1. Devices generate supplementary data for servers, and then send these data to servers secretly. By using the supplementary data, a server can process encrypted data from devices according to request of devices.
2. Devices send encrypted data to servers. In this way, devices can share their data with each other without leaking plaintext data to servers.
3. At some time, some device wants to get a result, which can be computed with data shared by devices. Then, it sends a request to servers.
4. According to the request sent by the device, a server can process encrypted data shared by devices with supplementary data and obtain a response. Then, the server sends its response to the request devices secretly.
5. If the request devices can collect a certain number of valid responses, it can obtain its desired result.
6. In this process, supplementary data and responses are verifiable. Moreover, by using the commitment technology, they can be publicly verifiable, and validators of blockchain can help users verify the related data.

Similar to the above scenario, the BeeKeeper 2.0 system works as follows:

1. One device executes a Setup algorithm to generate TX${}_{VK}$ and TX${}_{CS}$. TX${}_{VK}$ includes a verification key, while TX${}_{CS}$ includes encrypted core-shares (each encrypted core-share is encrypted by using a different server’s public-key) and commitments of core-shares. Then, the device sends these two transactions to the blockchain network.
2. Validators execute a VerifyTx_TX${}_{VK}$ algorithm to verify TX${}_{VK}$. TX${}_{VK}$ will be recorded in the blockchain iff VerifyTx_TX${}_{VK}$ outputs 1. Otherwise, the process returns to Step 1. Besides, the validators also execute a VerifyTx_TX${}_{CS}$ algorithm to verify TX${}_{CS}$. TX${}_{CS}$ will be recorded in the blockchain iff VerifyTx_TX${}_{CS}$ outputs 1. Otherwise, the process returns to Step 1.
3. Each server executes a CheckEnc_CS algorithm to check its encrypted core-share. The encrypted core-share is valid iff CheckEnc_CS outputs 1.
4. Each device executes an EncNum algorithm to generate TX${}_{EncN}$; then, it sends TX${}_{EncN}$ to the blockchain network.
5. Some device executes a Request algorithm to generate TX${}_{Requ}$; then, it sends TX${}_{Requ}$ to the blockchain network.
6. According to the transaction TX${}_{Requ}$, a server executes a Respond algorithm to generate a TX${}_{Resp}$, which includes an encrypted response (the response is encrypted by using the request device’s public-key) and this response’s commitment. Then, the server sends TX${}_{Resp}$ to the blockchain network.
7. The validators execute a VerifyTx_TX${}_{Resp}$ algorithm to verify TX${}_{Resp}$. TX${}_{Resp}$ will be recorded in the blockchain iff VerifyTx_TX${}_{Resp}$ outputs 1.
8. The request device executes a CheckEnc_Resp algorithm to check the encrypted responses recorded in the corresponding TX${}_{Resp}$s. An encrypted response is valid iff CheckEnc_Resp outputs 1.
9. When the request device has collected at least the threshold number of valid responses, it can execute a Recover algorithm to obtain the desired result.

BeeKeeper 2.0 has the following properties:

- Decentralization. Any peer of the blockchain can become a server of devices when desired by the peer and owner of the devices.
- Scalability. With more and more peers of the blockchain willing to act as servers, the overall processing power of the BeeKeeper 2.0 system gradually increases.
- Confidentiality. Servers cannot obtain any plaintext data.
- Full homomorphism. Servers can perform any-degree homomorphic multiplications and any number of additions on encrypted data according to the requests of devices.
- Public verifiability. Most verifiable data can be verified publicly. Therefore, the validators can help the devices and servers verify these publicly verifiable data. Moreover, these data can be verified efficiently. For instance, verifying a response of 10-degree polynomial of shared data takes approximately 0.0122 s; verifying a VK, which can be used to verify a response of 10-degree polynomial of shared data, takes approximately 1.33 s.
- Non-interactive. Servers can perform high-degree homomorphic multiplications and additions on encrypted numbers without interaction. Furthermore, verifiable data can be verified without interaction.
- Fault-tolerant. The BeeKeeper 2.0 system works as long as a threshold number of servers are honest and willing to serve devices.
- Lightweight. On the one hand, devices and servers do not need to maintain large storage since encrypted data are recorded in the blockchain. On the other hand, devices do not need a high-performance processor since computation on the data is performed by servers and the vast majority of verification work is performed by the validators of the blockchain.
- Data sharing. Devices can share their own data with other devices in a confidential way. Each device can utilize the whole data shared by other devices.

## 3. Background of Fully Homomorphic Non-Interactive Verifiable Secret Sharing

In our work, the main cryptographic primitive used is a fully homomorphic non-interactive verifiable secret (FHNVSS) sharing scheme [23]. We use the FHNVSS scheme to achieve a decentralized outsourcing computation (DOC) scheme, which is presented in Section 4. In this section, we briefly provide the basic background of the FHNVSS scheme. Details of the FHNVSS scheme can be found in Ref. [23].

A FHNVSS scheme includes a dealer, which is the data owner, and a certain number of servers. Concretely, the dealer confidentially shares numbers to servers by sending core-shares and encrypted numbers to the servers. When the dealer wants to obtain a result, which can be an any-degree polynomial of shared numbers, it sends a request to the servers. Then, the servers, without interaction, generate responses with encrypted numbers according to the request of the dealer. Once the dealer receives a threshold number of valid responses, it obtains the desired result. In this process, the VK, core-shares and responses are verifiable. Therefore, malicious participants and invalid data can be detected.

### 3.1. Data of FHNVSS

A FHNVSS scheme has the following six types of data.

- VK. The verification key (VK) is generated by the dealer. First, the validity of the VK can be verified publicly and used to verify the validity of the core-shares and responses.
- Core-shares. Core-shares are generated by the dealer and sent to the servers (each server will be given one set of core-shares).
- Encrypted numbers. Encrypted numbers are generated by the dealer and sent to the servers.
- Request. A request is generated by the dealer and sent to the servers.
- Responses. Responses are generated by the servers and sent to the dealer.
- Result. When the dealer collects a threshold number of valid responses, it can recover the desired result with these responses.

### 3.2. FHNVSS Algorithms

A FHNVSS scheme is a tuple of polynomial-time algorithms (A polynomial-time algorithm means that the time complexity of the algorithm is $O(n^{k})$, where *n* is the size of input of the algorithm):

(Setup, Ver_VK, Ver_CS, Requst, Gen_Resp, Ver_Resp, Recover).

- Setup($1^{\lambda }$, *t*, *n*, IDs)→ (VK, *n* core-shares)
  Upon input of a security parameter λ, the threshold *t*, the number of all shareholders *n* and all shareholders’ IDs, Setup randomly outputs a verification key (VK) and *n* core-shares for the *n* shareholders.
- Ver_VK(VK)→b
  Upon input of a VK, the *verifier* Ver_VK outputs b=1 iff the VK is valid.
- Ver_CS(core-share, ID, VK, sk)→b
  Upon input of a core-share, ID (a shareholder’s ID), VK and sk (a shareholder’s secret key), the *verifier* Ver_CS outputs b=1 iff the core-share is valid.
- Gen_Resp(core-share, request)→ a response
  Upon input of a core-share and a request, the *generator* Gen_Resp outputs a response.
- Ver_Resp(response, ID, VK, sk)→b
  Upon input of a response, ID (a shareholder’s ID), VK and sk (the dealer’s secret key), the *verifier* Ver_Resp outputs b=1 if the response is valid.
- Recover(responses, IDs)→ a result
  Upon input of the threshold number of responses and the senders’ IDs of these responses, Recover outputs a result.

### 3.3. Work Process of FHNVSS

We take a (t, n) FHNVSS scheme as an example to present the working process:

- Step 1: The dealer generates *n* sets of core-shares and a verification key (VK). Then, the dealer opens the VK. Anyone (including servers) can verify whether the VK is correctly computed by the dealer. If the VK is invalid, the dealer has to regenerate the core-shares and VK, else the participants join in the next step.
- Step 2: The dealer secretly sends *n* sets of core-shares to *n* servers. After receiving a core-share, a server can verify whether his core-share is valid via the VK. If the server’s core-share is invalid, he can ignore it and request that the dealer resends it.
- Step 3: The dealer encrypts secret numbers into encrypted numbers and then sends the encrypted numbers to the servers.
- Step 4: When the dealer needs to obtain a result, which could be an any-degree polynomial of secret numbers, it sends a request to *n* servers.
- Step 5: According to the request sent by the dealer, each active server will independently generate a response with its core-share (this process has no interaction with the other servers). Then, the server will send its response to the dealer securely.
- Step 6: After receiving the responses, the dealer can verify whether the responses are correctly computed by the corresponding servers. These verifications do not require interaction with the servers. If a response is invalid, the dealer can ignore the response or request that the corresponding server resend a response. Finally, the dealer can recover the desired result if it collected at least *t* valid responses.

## 4. Decentralized Outsourcing Computation Scheme

In this section, we present a decentralized outsourcing computation (DOC) scheme based on blockchain and the FHNVSS scheme [23]. A basic instance of the DOC scheme includes a dealer, a certain number of servers and validators of the blockchain. In this instance, the dealer and servers execute a FHNVSS scheme by sending transactions to the blockchain. The validators check all transactions, and only transactions accepted by the validators can be recorded in the blockchain. Specifically, the dealer uses transactions to publish the verification key (VK), send encrypted core-shares to servers and publish encrypted numbers to servers; servers use transactions to send encrypted responses to the dealer; and the validators of the blockchain check all these transactions before they are recorded in the blockchain. In the following, we present all data structures, algorithms and a detailed work process of the DOC.

### 4.1. Data Structure

A DOC scheme has the following ten types of data structures:

Ledger. The DOC scheme can be applied on top of any blockchain (ledger) platform that is a sequence of transactions, such as Bitcoin, Ethereum and Hyperledger Fabric. At any given time *T*, all users have access to $L_{T}$, that is, the ledger at time *T*. The ledger is append-only (i.e., $T<T^{\prime }$ implies that $L_{T}$ is a prefix of $L_{T^{\prime }}$). The transactions in the ledger include Basecoin transactions and new types of transaction that are described below.

Address. Each peer in the blockchain network can generate an arbitrary number of key pairs. In the DOC scheme, a key pair is (pk, sk), where pk=skG (*G* is a base point of ECC [24] and sk is a 256-bit random number). Furthermore, pk is also an address (ID).

Commitment. We use the point multiplication of ECC to compute a commitment of a private value as follows:

commitment=aG,

where *a* is the private value and *G* is a base point of ECC.

Signature. We use a digital signature scheme $\mathrm{Sig}=\left(S_{sig},\;V_{sig}\right)$:

- S${}_{sig}$(sk,m)→σ. Given a secret key sk and a message *m*, S${}_{sig}$ signs *m* to obtain a signature σ.
- V${}_{sig}\left(pk,\;\sigma ,m\right)\to b$. Given a public-key pk, message *m* and signature σ, V${}_{sig}$ outputs b=1 iff the signature σ is valid for *m*; else, it outputs b=0.

Public-key encryption. We use a public-key encryption scheme Enc = (E${}_{enc}$, D${}_{enc}$):

- E${}_{enc}$(pk,m)→c. Given a public-key pk and a message *m*, E${}_{enc}$ encrypts *m* to obtain a ciphertext *c*.
- D${}_{enc}\left(sk,\;c\right)\to m$. Given a secret key sk and ciphertext *c*, D${}_{enc}$ outputs a message *m*.

The components of the DOC scheme include a dealer and a certain number of servers. The verification key, core-share, request, response and result are generated by the dealer and servers. The details of these five types of data can be found in Ref. [23]. In the following, we briefly present these data structures.

**Verification key.** A verification key (VK) is a set of commitments of coefficients of polynomials that are randomly sampled by the dealer, and the VK is available to all users in the DOC scheme. A VK is generated by the dealer at the “start time” and can be used in all verification algorithms.

**Core-shares.** When calculating the VK, the dealer randomly samples a set of polynomials. Then, it inputs the ID of each server into these polynomials to generate a core-share for this server. Therefore, a core-share is a set of random numbers in $F_{q}$, where *q* is the prime order of *G*.

**Request.** A request is a polynomial as follows:

$$a_{1}^{2}a_{2}^{3}+a_{3}^{5}+a_{4}a_{5}^{3}+a_{6}^{4}+a_{7}^{3}+a_{8}a_{9}a_{10},$$

where $a_{1},\;a_{2},\;\ldots ,\;a_{10}$ are symbols. $a_{1},\;a_{2},\;\ldots ,\;a_{10}$ corresponds to 10 plaintext numbers that do not reveal plaintext numbers. According to this polynomial, the servers will know how to process their encrypted numbers.

**Response.** After seeing a request sent by the dealer, a server will generate its response to this request, as well as its core-share and encrypted numbers. A response is a number in $F_{q}$.

**Result.** After receiving a threshold number of valid responses, the dealer will use Lagrangian interpolation to recover the desired result, which is a number in $F_{q}$.

**New transactions.** These five types of data will be recorded in the payload of transactions. Therefore, a DOC scheme has five new types of transactions (TX${}_{VK}$, TX${}_{CS}$, TX${}_{EncN}$, TX${}_{Requ}$, and TX${}_{Resp}$).

- TX${}_{VK}$. A verification key transaction TX${}_{VK}$ includes only a verification key (VK).
- TX${}_{CS}$. A core-share transaction TX${}_{CS}$ is a tuple (${\mathrm{cmcs}}_{1}$, ${\mathrm{cmcs}}_{2}$, …,${\mathrm{cmcs}}_{n}$, ${\mathrm{encs}}_{1}$, ${\mathrm{encs}}_{2}$, …,${\mathrm{encs}}_{n}$), where ${\mathrm{cmcs}}_{i}$ is the commitments of the core-share of the *i*th server and ${\mathrm{encs}}_{i}$ is the encrypted core-share of the *i*th server, i=1, 2, …, n.
- TX${}_{EncN}$. An encrypted numbers transaction TX${}_{EncN}$ includes a set of encrypted numbers.
- TX${}_{Requ}$. A request transaction TX${}_{Requ}$ includes a request.
- TX${}_{Resp}$. A response transaction TX${}_{Resp}$ is a tuple (cmresp, enresp), where cmresp is a commitment of a response and where enresp is an encrypted response.

### 4.2. Algorithms

A DOC scheme is a tuple of polynomial-time algorithms:

(Setup, EncNum, Request, Respond, Recover, VerifyTx, CheckEnc)

with the following syntax and semantics.

- Setup($1^{\lambda }$, *t*, *n*, IDs)→ (VK, *n* core-shares, TX${}_{VK}$, TX${}_{CS}$).
  Upon input of a security parameter λ, the threshold *t*, the number of all servers *n* and the IDs of the servers, Setup randomly outputs a verification key (VK), *n* core-shares of *n* servers, TX${}_{VK}$ and TX${}_{CS}$. The Setup algorithm is executed by the dealer and is not reused until the dealer wants to restart the DOC scheme. TX${}_{VK}$ includes VK, while TX${}_{CS}$ includes commitments of core-shares and encrypted core-shares.
- EncNum(numbers, key secret)→ (encrypted numbers, TX${}_{EncN}$).
  Upon input of numbers and the key secret, EncNum outputs encrypted numbers and TX${}_{EncN}$. The EncNum algorithm is executed by the dealer. TX${}_{EncN}$ includes VK encrypted numbers.
- Request(request)→ (TX${}_{Requ}$). Upon input of a request, Request outputs a TX${}_{Requ}$. The Request algorithm is executed by the dealer. TX${}_{EncN}$ includes only a request.
- Respond(request, encrypted numbers, core-share)→ (response, TX${}_{Resp}$).
  Upon input of a request, encrypted numbers, and core-share, Respond outputs a response and TX${}_{Resp}$. The Respond algorithm is executed by the servers. TX${}_{Resp}$ is a tuple (cmresp,enresp), where cmresp is a commitment of a response and enresp is the encrypted response.
- Recover(*t* responses, *t* IDs)→ (result). s Upon input of *t* responses and *t* IDs, Recover outputs a result. The Recover algorithm is executed by the dealer.

**Verify transactions.** The algorithm VerifyTx is used to check the validity of the commitments in a transaction, and it has three variants VerifyTx_TX${}_{VK}$, VerifyTx_TX${}_{CS}$ and VerifyTx_TX${}_{Resp}$), which are executed by the validators of the blockchain. Only transactions with valid commitments can be recorded in the blockchain. VerifyTx_TX${}_{VK}$ checks the validity of the VK in the TX${}_{VK}$; VerifyTx_TX${}_{CS}$ uses the VK to check the validity of the commitments of the core-shares in the TX${}_{CS}$; and VerifyTx_TX${}_{Resp}$ uses the VK to check the validity of the commitment of the response in the TX${}_{Resp}$. The three algorithms output a bit *b*: b=1 iff the corresponding transaction’s commitments are valid.

**Check encrypted messages.** The CheckEnc algorithm checks the encrypted messages in a transaction payload, and it has two variants: CheckEnc_CS and CheckEnc_Resp. CheckEnc_CS is executed by the servers to check whether the commitment of a plaintext core-share equals the commitment of the core-share recorded in the TX${}_{CS}$. CheckEnc_Resp is executed by the dealer to check whether the commitment of a plaintext response equals the commitment of the response recorded in the TX${}_{Resp}$. The two algorithms output a bit *b*:*b* = 1 iff the commitment of plaintext data equals the commitment of data recorded in the corresponding transaction.

**Remark** **1.** *The security of DOC is based completely on the FHNVSS scheme [23]. The security analysis of the FHNVSS scheme can be found in Ref. [23].*

### 4.3. Construction of a Decentralized Outsourcing Computation Scheme

**Figure 2:**
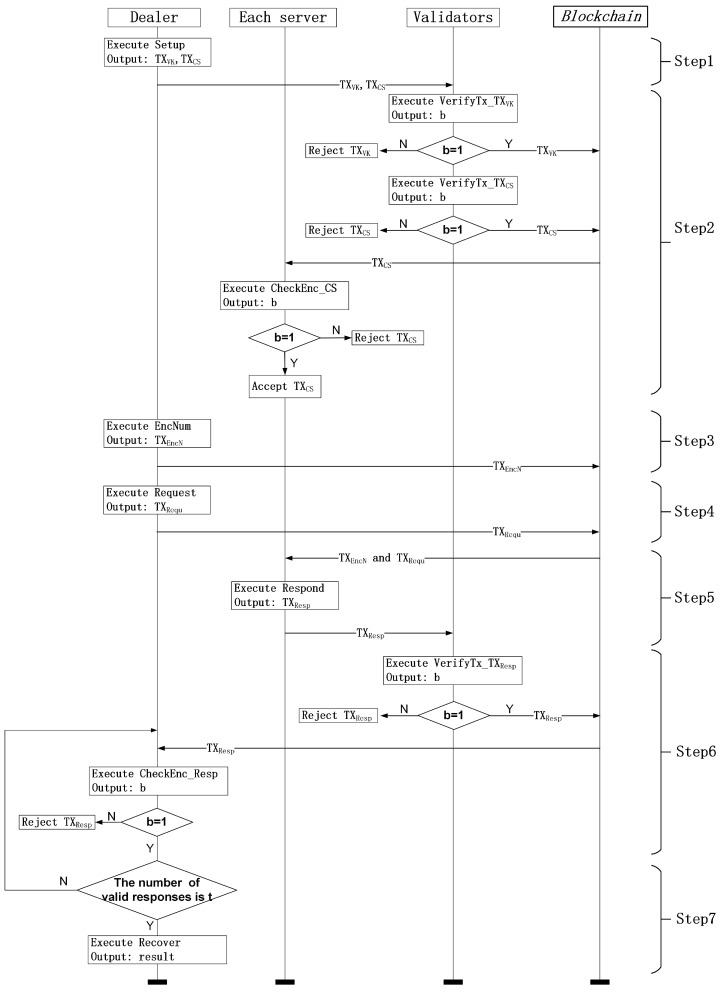
Construction of a decentralized outsourcing computation.

In this subsection, we construct a (t, n) decentralized outsourcing computation (DOC) scheme, which includes n+1 clients and a certain number of validators of the blockchain. Specifically, one of these n+1 clients acts as a dealer, and the other *n* clients act as servers. In the following, the work process of DOC scheme is presented, and it is also illustrated in Figure 2.

- Step 1: The dealer executes the Setup algorithm to obtain two transactions TX${}_{VK}$ and TX${}_{CS}$ and then sends these two transactions to the validators.
- Step 2:
  – The validators execute algorithms VerifyTx_TX${}_{VK}$ and VerifyTx_TX${}_{CS}$ to verify TX${}_{VK}$ and TX${}_{CS}$, respectively. If VerifyTx_TX${}_{VK}$ outputs 1, the validators accept TX${}_{VK}$. If VerifyTx_TX${}_{CS}$ outputs 1, the validators accept TX${}_{CS}$. The transactions accepted by the validators will be recorded in the blockchain.
  – After TX${}_{CS}$ is recorded in the blockchain, each server executes the CheckEnc_CS algorithm to check the encrypted core-share in TX${}_{CS}$. If CheckEnc_CS outputs 1, the server accepts this encrypted core-share in TX${}_{CS}$.
- Step 3: The dealer executes the EncNum algorithm to obtain a transaction TX${}_{EncN}$ and sends this transaction to the blockchain network.
- Step 4: The dealer executes the Request algorithm to obtain a transaction TX${}_{Requ}$ and sends this transaction to the blockchain network.
- Step 5: According to TX${}_{Requ}$, each server executes the Respond algorithm to obtain a transaction TX${}_{Resp}$ and sends this transaction to the validators.
- Step 6:
  – The validators execute the algorithm VerifyTx_TX${}_{Resp}$ to verify TX${}_{Resp}$. If VerifyTx_TX${}_{Resp}$ outputs 1, the validators accept TX${}_{Resp}$, and the accepted TX${}_{Resp}$ will be recorded in the blockchain. Otherwise, the validators will reject it.
  – After TX${}_{Resp}$ is recorded in the blockchain, the dealer executes the CheckEnc_Resp algorithm to check the encrypted response in TX${}_{Resp}$. If CheckEnc_Resp outputs 1, the dealer accepts this encrypted response in TX${}_{Resp}$.
- Step 7: When the dealer receives *t* valid responses, the Recover algorithm is executed to obtain the desired result.

**Remark** **2.** *In this work, we hope to achieve that data recorded in the blockchain are credible. Therefore, publicly verifiable data (e.g., verification key, commitments of core-shares and responses, and encrypted numbers) should be stored in the blockchain and be verified by validators. These data can provide the credibility of the system. Thereby, these data are stored in the blockchain in this manuscript. Indeed, this will add certain burden of system storage since the storage space is one of the main limitations of blockchain. However, we know that the main part of the storage space is used by the encrypted numbers. Thereby, we can solve the storage issue by modifying the working mode. For instance, the dealer sends the encrypted numbers to servers off-chain, and it only stores a hash value of the encrypted numbers in the blockchain. In this way, servers can also check the integrity of encrypted numbers received, which reduces the storage burden of the system. Besides, validators cannot help the dealer verify the correctness of responses, and the dealer has to perform the response verification by itself. This modified working mode makes a compromise between credibility and storage issue.*

## 5. BeeKeeper 2.0

In this section, we first instantiate all the building blocks of DOC; then, the instantiated DOC scheme is deployed in the IoT scenario to achieve a confidential blockchain-enabled IoT system, called BeeKeeper 2.0. In BeeKeeper 2.0, the dealer is replaced by a group of devices, and the devices can share their data with each other and use other devices’ data in a confidential manner. The servers can help the devices perform fully homomorphic computations on encrypted data from other devices according to the request of a device, but they cannot obtain any plaintext data.

### 5.1. Instantiation of Building Blocks

In this subsection, we instantiate all the building blocks of the DOC scheme. These building blocks are used in the BeeKeeper 2.0 system.

**Instantiating large number computations.** Algorithms Setup, EncNum, Request, Respond and Recover are all executed with 256-bit large number computation and a 256-bit large prime field. We utilized the Python GNU Multiple Precision (GMP) Arithmetic Library [25] to calculate the large number computations of Recover.

**Instantiating commitments.** Algorithms Setup, Respond VerifyTx_TX${}_{VK}$, VerifyTx_TX${}_{CS}$, VerifyTx_TX${}_{Resp}$, CheckEnc_CS and CheckEnc_Resp either compute with commitments or generate commitments. These commitments are the results of point multiplications of ECC. We used the high-speed Pairing-Based Cryptography (PBC) library [26] to compute the point multiplications of ECC.

**Instantiating pairing.** We also used the high-speed PBC library [26] to compute pairing. TX${}_{VK}$ has a set of commitments as follows:

$$CM_{s},\;CM_{s^{2}},\;CM_{s^{3}},\;\ldots ,\;CM_{s^{k}},$$

where $CM_{s},\;CM_{s^{2}},\;CMs^{3},\;\ldots ,\;CM_{s^{k}}$ are points of ECC generated by *G*, which is the base point of ECC. $CM_{s},\;CM_{s^{2}},\;CMs^{3},\;\ldots ,\;CM_{s^{k}}$ are commitments of $s,\;s^{2},\;s^{3},\;\ldots ,\;s^{k}$ and no one can obtain $s,\;s^{2},\;s^{3},\;\ldots ,\;s^{k}$ from $CM_{s^{2}},\;CMs^{3},\;\ldots ,CM_{s^{k}}$. The validity of $CM_{s},\;CM_{s^{2}},\;CMs^{3},\;\ldots ,\;CM_{s^{k}}$ is verified by using pairing e(·, ·). Specifically, $CM_{s^{2}},\;CMs^{3},\;\ldots ,CMs^{k}$ are valid iff the following formulas hold.

$$e\left(CM_{s},\;CM_{s}\right)=e\left(CM_{s^{2}},\;G\right),\;e\left(CM_{s},\;CM_{s^{2}}\right)=e\left(CM_{s^{3}},\;G\right),\;\ldots ,\;e\left(CM_{s},\;CM_{s^{k-1}}\right)=e\left(CM_{s^{k}},\;G\right).$$

Next, we explain why the above equations verify the validity of $CM_{s},\;CM_{s^{2}},\;CMs^{3},\;\ldots ,\;CM_{s^{k}}$. Let the hidden value of $CM_{s}$ be *s*. If $e\left(CM_{s},\;CM_{s}\right)=e\left(CM_{s^{2}},\;G\right)$, it demonstrates that hidden value of $CM_{s^{2}}$ is $s^{2}$; if $e\left(CM_{s},\;CM_{s^{2}}\right)=e\left(CM_{s^{3}},\;G\right)$, it demonstrates that hidden value of $CM_{s^{3}}$ is $s^{3}$; etc. Similarly, if $e\left(CM_{s},CM_{s^{k-1}}\right)=e\left(CM_{s^{k}},\;G\right)$, it demonstrates that hidden value of $CM_{s^{k}}$ is $s^{k}$.

**Instantiating Signature.** Every transaction should include the signature of the transaction’s generator. A transaction with an incorrect signature is invalid. We used secp256k1-based, which is an elliptic curve, and ECDSA [27] as the signature scheme Sig to sign transactions.

**Instantiating Encryption.** TX${}_{CS}$ and TX${}_{Resp}$ include some encrypted core-shares and responses. We used secp256k1-based ECIES [28], which is a public-key encryption scheme, as the encryption scheme Enc to encrypt the core-shares and responses. With this encryption scheme, only the holder of the corresponding secret key can decrypt the encrypted data.

**Instantiating the hash function.** We used SHA-256 [29] as the hash function H to compute the hash value.

**Instantiating the DOC scheme.** We implemented all algorithms of the DOC scheme in Python on a Linux server with 16 GB RAM and a 3.1 GHz processor. Moreover, we deployed the instantiation of the DOC on the Hyperledger Fabric blockchain to construct the BeeKeeper 2.0 system as a blockchain-enabled intelligent lightweight IoT system.

### 5.2. Construction of BeeKeeper 2.0

A basic instance of the BeeKeeper 2.0 system includes a group of devices and a certain number of servers. Specifically, in a basic instance of BeeKeeper 2.0, once the devices have been set, they will work automatically. In the following, we consider a basic instance to present the work process of the BeeKeeper 2.0 system. We omit the verification process since it is the same as that of DOC.

**Setup.** The devices select one device to execute the Setup algorithm to generate transactions TX${}_{VK}$ and TX${}_{CS}$, and this selected device sends these two transactions to the blockchain network. If the devices want to update TX${}_{VK}$ and TX${}_{CS}$, they reselect a device re-execute the Setup algorithm.

**Share data.** Each device can execute the EncNum algorithm to generate TX${}_{EncN}$ and send this transaction to the blockchain network. This device can execute the EncN algorithm as many times as desired. All devices can independently and continuously run the EncNum algorithm to share their data with each other in this way.

**Request and respond.** Each device can execute the Request algorithm to generate TX${}_{Requ}$, which is used to let the servers help the devices perform homomorphic computations on the encrypted data shared by all devices. Then, the device sends this transaction to the blockchain network. Next, servers execute the Respond algorithm to generate TX${}_{Resp}$ according to the requests recorded in the TX${}_{Requ}$. At that time, they sends their own TX${}_{Resp}$s to the blockchain network. Each device can execute Request as many times as desired. Each device can independently and continuously use the encrypted data shared by all devices in this method.

**Recover.** When a device has received at least the threshold number of valid responses, the Recover algorithm is executed to recover the desired result.

In practical applications, algorithms belong to different participants (dealer and servers). The affiliation of these functions is shown in Table 1.

## 6. Performance Evaluation

In this section, we present a performance evaluation of the BeeKeeper 2.0 system by deploying the system on the Hyperledger Fabric blockchain. Specifically, we deploy the BeeKeeper 2.0 system and Hyperledger Fabric on four Ubuntu 16.04 environment servers with 2 GB RAM and a 1 GHz processor. A series of measurements are made with respect to the blockchain performance and algorithm performance. The theoretical throughput of Hyperledger is about 2000 TPS (TPS denotes transactions per second) in actual production environment. Limited by the testing environment, the testing system’s throughput can only reach about 100 TPS, so we simply measured the success rate of the system as an example of what would happen when the invoke concurrency is fast outpacing the throughput of the blockchain. Once the system is deployed to production environment, no transactions would fail unless the concurrency is beyond the processing rate of the blockchain. In the following, the performance of the algorithms is presented.

### 6.1. Performance of the BeeKeeper 2.0 Algorithms

In our experiments, we deployed a (3,4) BeeKeeper 2.0 system on our could servers, where (3,4) means that there are four servers and that the desired result can be recovered with at least three valid responses sent by the servers. We implemented all the algorithms of BeeKeeper 2.0 in Python. In this implementation, the high-speed Pairing-Based Cryptography (PBC) library [26] was used to compute the point multiplication of ECC and pairing; the GNU Multiple Precision (GMP) Arithmetic Library [25] was utilized to calculate the large number computations; and SHA-256 was used as the hash function. All large number computations were computed over a 256-bit large prime number that is the order of the base point of ECC. We tested the performance of all the algorithms by varying the maximum degree of the polynomial that the servers can calculate from 4 to 10. The performance of the algorithms is illustrated in Table 2.

### 6.2. Large-Scale Network Simulation

In terms of the blockchain performance, we used Hyperledger Fabric [22] as the carrier of the BeeKeeper 2.0 system and used Hyperledger Caliper [30] for performance testing. The algorithms of BeeKeeper 2.0 were invoked in the blockchain. Because the throughput of Hyperledger Fabric on our test environment is nearly 100 TPS, we performed high-concurrency performance testing to simulate a high TPS scenario.

The main indicators include *chaincode invoke latency, success rate, query latency and send rate*. Specifically, *chaincode invoke latency* denotes the latency in a transaction being uploaded in the blockchain; *success rate* refers to the probability that the transactions were successfully recorded in the blockchain; *query latency* is the latency for a peer to query the blockchain; and *send rate* (TPS) denotes the number of transactions sent by peers per second.

In our tests, we varied the send rate from 50 to 300 TPS to observe how the other indicators change with the send rate. We then analyzed the efficiency of the system based on the results of these measurements.

**Figure 3:**
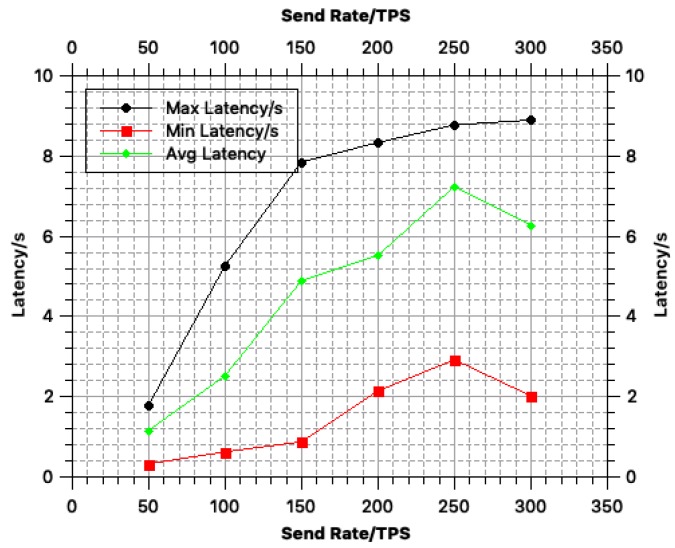
Chaincode invoke latency.

The change in the *chaincode invoke latency* with the send rate is illustrated in Figure 3. According to Figure 3, the average chaincode invoke rate gradually increases as the send rate increases. When the send rate reaches 300 TPS, the chaincode invoke latency decreases slightly.

**Figure 4:**
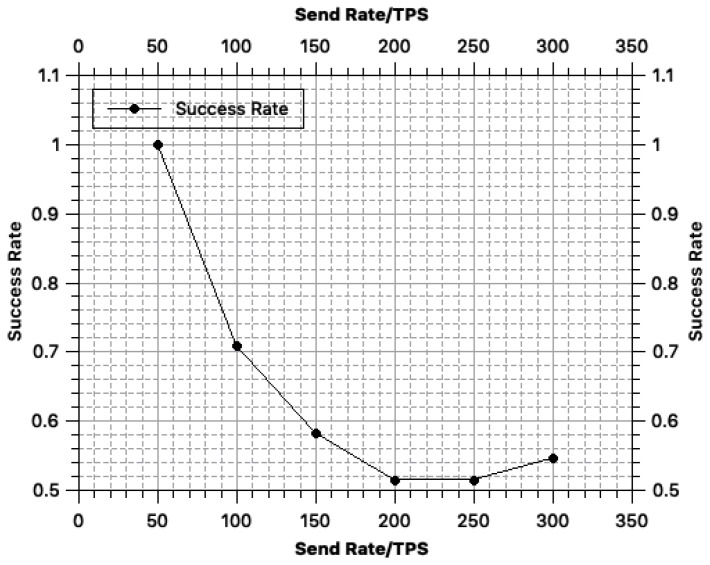
Transaction success rate.

The change in the *success rate* with the send rate is illustrated in Figure 4. According to Figure 4, the average success rate gradually decreases as the send rate increases. When the send rate is 300 TPS, the success rate increases slightly.

The success rate shown in Figure 4 is the success rate measured around the bottleneck of the cluster’s total throughput. The bottleneck of a cluster’s bottleneck will vary with the processing speed of each server and the size of the entire cluster. If it can be deployed in a large enough cluster, throughput would theoretically increase to 2000 TPS. However, limited by the environment, we deployed on four dual-core cloud servers and measured that the throughput is about 100 TPS, so we selected several sets of send rates around the throughput bottleneck to show what would happen when the send rate fast outpaces the bottleneck of throughput. Once the system is deployed in the production environment, as long as the throughput bottleneck (i.e., around 2000 TPS) is not exceeded, the success rate of the algorithm execution will always be 100%.

**Figure 5:**
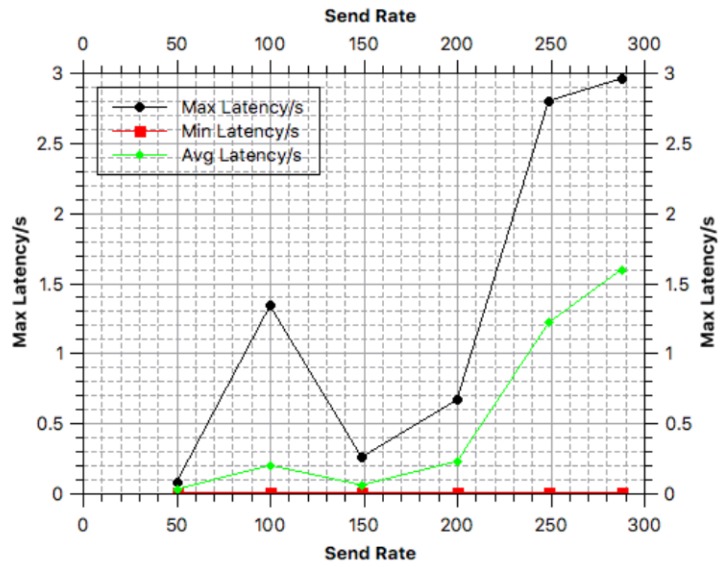
Query latency.

The change in the *query latency* with the send rate is illustrated in Figure 5. According to Figure 5, the query average latency gradually increases as the send rate increases. The query speed is less affected by the throughput, and the success rate is stable at 100%. Therefore, we take the reading speed of 2.83 s measured at 500 TPS.

In our test environment, the throughput (send rate) of the system is approximately 100 TPS. According to our test results in Figure 3, Figure 4 and Figure 5, when the throughput reaches 100 TPS: (i) the average transaction time by the blockchain is approximately 2.51 s; (ii) the average success rate of transactions being recorded in the blockchain is approximately 73%; and (iii) the query latency is approximately 0.2 s. However, once the system be deployed in the real-world environment, the success rate of it would constantly be 100%.

**Figure 6:**
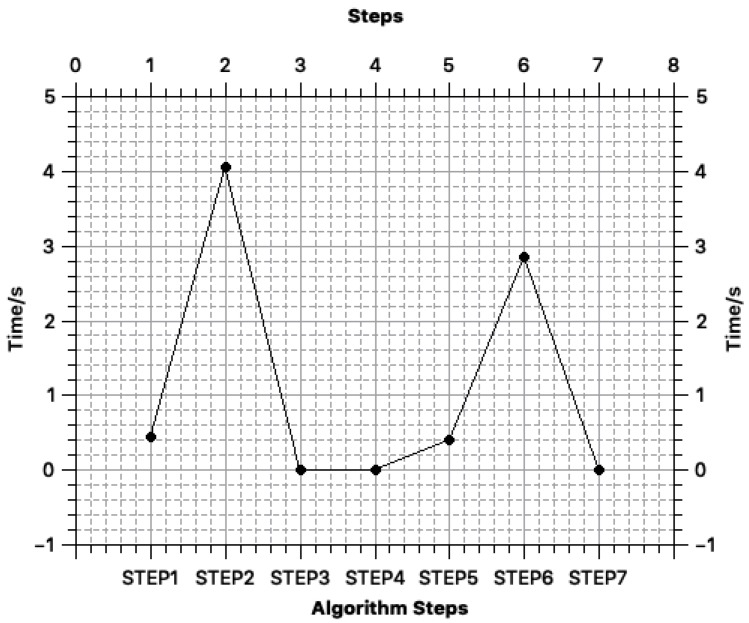
Step latency.

To analyze the time consumed by each step of BeeKeeper 2.0 in practical applications, we deployed a (3,4) BeeKeeper 2.0 system in our test environment. Furthermore, we set the largest degree of polynomial that the servers can process to 10. Moreover, to ensure an adequate success rate for satisfactory user experience and operation in practice, we set the initial value of throughput to 100 TPS since the test environment throughput of our system is approximately 100 TPS. Therefore, BeeKeeper 2.0 includes seven steps, as mentioned in Figure 2, since the BeeKeeper 2.0 system process is similar to that of a DOC scheme. The time consumption of the steps in the actual process is shown in Figure 6. In the case that the network is not blocked, the processing speed is approximately 2.51 s, and the total time from the begin to the end of the process is less than 2.94 s, which is completely acceptable and even improves upon the processing speed of the current blockchain. Therefore, the encryption computing power required by this protocol can also be borne by a mobile terminal, and it is highly adaptable. If the blockchain is completely used as a medium for data sharing instead of as a smart contract platform, a small hash rate is required for an endorser node.

According to Figure 6, the protocol steps with the greatest time consumption in the whole test process are Steps 2 and 6 (4.2 s and 2.9 s, respectively), and the cost of these steps is allocated to the preparation stage and verification process before the on-chain operations. These two steps have no effect on obtaining a result quickly. Step 4 is the request stage; Steps 5 and 6 are the respond stage; and Step 7 is the recover stage. According to Figure 6, the time consumed between sending a request and recovering the final result is approximately 3.3 s. Therefore, the processing speed on the chain can theoretically reach 3.3 s. For the BeeKeeper 2.0 system, this speed will not result in any additional burden or delay, thus the actual test of the IoT blockchain system theoretically satisfies the production needs.

## 7. Conclusions

We studied a blockchain-enabled IoT system. First, we proposed a decentralized outsourcing computation (DOC) scheme, where servers can perform homomorphic computations on encrypted data and users can verify whether the servers are honest. Second, we applied the DOC scheme in an IoT scenario to achieve a blockchain-enabled intelligent lightweight IoT system, called BeeKeeper 2.0. In the BeeKeeper 2.0 system, devices can share their own data without leaking plaintext data to the servers, and devices can use the data shared by all devices by sending requests to the servers. Finally, we conducted a detailed performance evaluation of the BeeKeeper 2.0 system. Specifically, we deployed the BeeKeeper 2.0 system on the Hyperledger Fabric blockchain and used the Hyperledger Caliper for performance testing. According to our tests, when the servers can process at most 10-degree polynomials of shared numbers with encrypted numbers, the time consumed between the request stage and the recover stage is approximately 3.3 s, which theoretically satisfies production needs.

## Figures and Tables

**Table 1:** Affiliation of algorithms.

Participant	Algorithms of Participant
Device	Setup,EncNum,Request,Recover,CheckEnc_TX${}_{Resp}$
Server	Respond,CheckEnc_TX${}_{CS}$
Validator	VerifyTx_TX${}_{VK}$,VerifyTx_TX${}_{CS}$,VerifyTx_TX${}_{Resp}$

**Table 2:** Performance of algorithms.

*k*	4	5	6	7	8	9	10
Setup	0.1282777	0.1560096	0.2025866	0.2608900	0.3187592	0.3766045	0.4376585
EncNum	0.0000354	0.0000364	0.0000366	0.0000352	0.0000358	0.0000362	0.0000362
Request	0.0000042	0.0000043	0.0000043	0.0000044	0.0000043	0.0000044	0.0000043
Respond	0.0019154	0.0017049	0.0017740	0.0017232	0.0016155	0.0017323	0.0017263
Recover	0.0004520	0.0003650	0.0003395	0.0003912	0.0004041	0.0003230	0.0003862
VerifyTx ${}_{VK}$	0.1483874	0.2377457	0.3686747	0.5367989	0.7667174	1.0569477	1.3313813
VerifyTx ${}_{CS}$	0.0030455	0.0046279	0.0059521	0.0074007	0.0105726	0.0123372	0.0142269
VerifyTx ${}_{Resp}$	0.0238838	0.0321993	0.0496330	0.0658040	0.0818657	0.0965642	0.1221394
CheckEnc_CS	0.0012397	0.0012423	0.0012381	0.0012378	0.0012473	0.0012376	0.0012333
CheckEnc_Resp	0.0012635	0.0012635	0.0012635	0.0012635	0.0012635	0.0012635	0.0012635

***k*** denotes the maximum degree of the polynomial that the server can calculate. We implemented a (3,4) BeeKeeper 2.0 system to show the performance of algorithms.
